# 1-Methyl-2-methyl­sulfanyl-6-nitro-1*H*-benzimidazole

**DOI:** 10.1107/S1600536814004723

**Published:** 2014-03-08

**Authors:** Mohamed El Ghozlani, El Mostapha Rakib, Abdelouahid Medaghri-Alaoui, Mohamed Saadi, Lahcen El Ammari

**Affiliations:** a Laboratoire de Chimie Organique et Analytique, Université Sultan Moulay Slimane, Faculté des Sciences et Techniques, Béni-Mellal, BP 523, Morocco; b Laboratoire de Chimie du Solide Appliquée, Faculté des Sciences, Université Mohammed V-Agdal, Avenue Ibn Battouta, BP 1014, Rabat, Morocco

## Abstract

The mol­ecule of the title compound, C_9_H_9_N_3_O_2_S, is built up from fused five- and six-membered rings connected to methyl­sulfanyl and nitro groups, respectively. The mean plane through the fused ring system is inclined slightly relative to the plane passing through the nitro group [dihedral angle = 3.6 (2)°]. In the crystal, mol­ecules are linked by C—H⋯O hydrogen bonds and π–π inter­actions between imidazole rings [inter-centroid distance = 3.667 (3) Å], forming a three-dimensional network.

## Related literature   

For the biological activity of benzimidazoles, see: Achar *et al.* (2010 ▶); Boiani & Gonzalez (2005 ▶); Ishida *et al.* (2006 ▶); Kamal *et al.* (2008 ▶); Kus *et al.* (2004 ▶); LaPlante *et al.* (2004 ▶).
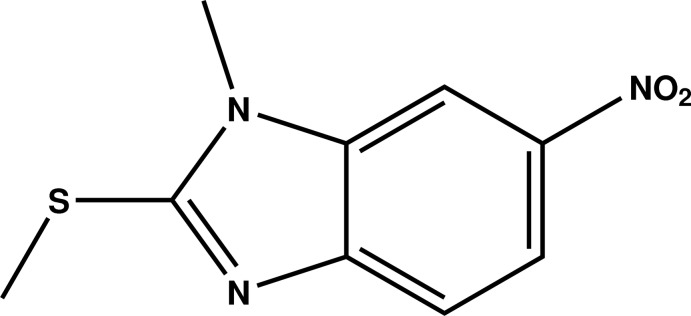


## Experimental   

### 

#### Crystal data  

C_9_H_9_N_3_O_2_S
*M* *_r_* = 223.25
Monoclinic, 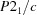

*a* = 11.7213 (4) Å
*b* = 11.8991 (4) Å
*c* = 7.3025 (3) Å
β = 103.523 (1)°
*V* = 990.26 (6) Å^3^
*Z* = 4
Mo *K*α radiation
μ = 0.31 mm^−1^
*T* = 296 K
0.42 × 0.31 × 0.26 mm

#### Data collection  

Bruker X8 APEX diffractometer
Absorption correction: multi-scan (*SADABS*; Bruker, 2009 ▶) *T*_min_ = 0.658, *T*_max_ = 0.746
11751 measured reflections
2772 independent reflections
2350 reflections with *I* > 2σ(*I*)
*R*_int_ = 0.025

#### Refinement  

*R*[*F* ^2^ > 2σ(*F* ^2^)] = 0.039
*wR*(*F* ^2^) = 0.116
*S* = 1.07
2772 reflections
136 parameters
H-atom parameters constrained
Δρ_max_ = 0.34 e Å^−3^
Δρ_min_ = −0.19 e Å^−3^

### 

Data collection: *APEX2* (Bruker, 2009 ▶); cell refinement: *SAINT* (Bruker, 2009 ▶); data reduction: *SAINT*; program(s) used to solve structure: *SHELXS97* (Sheldrick, 2008 ▶); program(s) used to refine structure: *SHELXL97* (Sheldrick, 2008 ▶); molecular graphics: *ORTEP-3 for Windows* (Farrugia, 2012 ▶); software used to prepare material for publication: *PLATON* (Spek, 2009 ▶) and *publCIF* (Westrip, 2010 ▶).

## Supplementary Material

Crystal structure: contains datablock(s) I. DOI: 10.1107/S1600536814004723/tk5298sup1.cif

Structure factors: contains datablock(s) I. DOI: 10.1107/S1600536814004723/tk5298Isup2.hkl

Supporting information file. DOI: 10.1107/S1600536814004723/tk5298Isup3.cml

CCDC reference: 989365

Additional supporting information:  crystallographic information; 3D view; checkCIF report

## Figures and Tables

**Table 1:** Hydrogen-bond geometry (Å, °)

*D*—H⋯*A*	*D*—H	H⋯*A*	*D*⋯*A*	*D*—H⋯*A*
C8—H8*A*⋯O1^i^	0.96	2.53	3.393 (2)	150
C3—H3⋯O2^ii^	0.93	2.65	3.3038 (19)	128
C9—H9*A*⋯O2^iii^	0.96	2.67	3.563 (2)	155

Symmetry codes: (i) 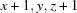; (ii) 
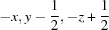
; (iii) 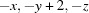.

**Fig. 1.**
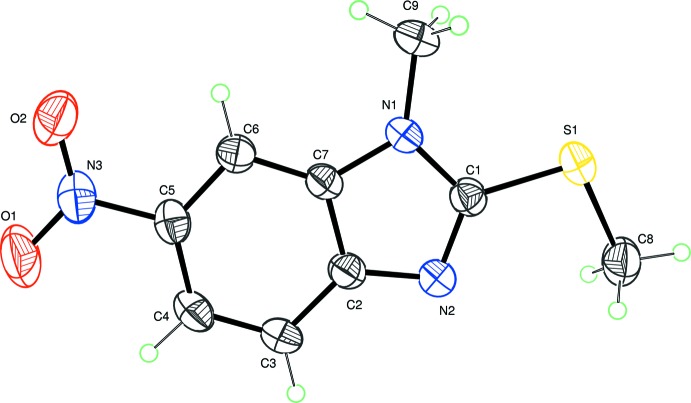
Plot of the molecule of the title compound with the atom-labelling scheme. Displacement ellipsoids are drawn at the 50% probability level. H atoms are represented as small circles.

**Fig. 2.**
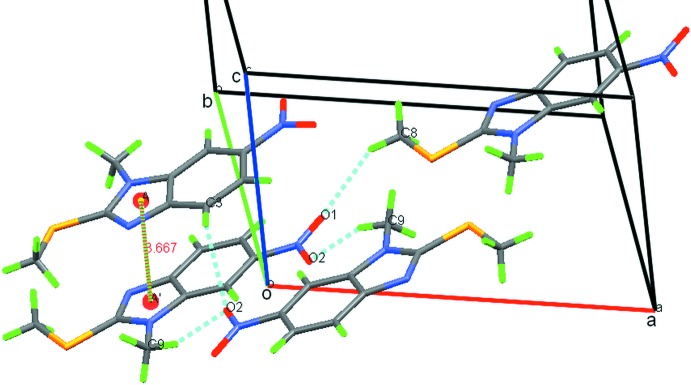
Partial crystal packing for the title compound showing molecules linked by π–π interactions and hydrogen bonds (dashed lines).

## supplementary crystallographic information

### 2. Structural commentary

Benzimidazole derivatives are of wide inter­est because of their diverse biological activities and clinical applications. This fused-ring system exhibits a broad spectrum of biological activities, that is, anti-cancer, anti-viral, anti-bacterial, anti-inflammatory, anti-oxidant and anti-leukaemic (LaPlante *et al.*, 2004; Ishida *et al.*, 2006; Boiani & Gonzalez, 2005; Achar *et al.*, 2010; Kus *et al.*, 2004; Kamal *et al.*, 2008).

The molecule of the title compound, C_9_H_9_N_3_O_2_S, is formed by a fused five- and six-membered rings as shown in Fig.1. The mean plane through the fused ring system (N1,N2,C1 to C7) is slightly inclined relative to the mean plane passing through the nitro group with a dihedral angle of 3.6 (2)°. In the crystal, molecules are linked by C—H···O hydrogen bonds and π–π inter­actions between indazole rings [inter-centroid distance = 3.667 (3) Å], forming a three-dimensional network as shown in Fig.2 and Table 1.

### 5. Synthesis and crystallization

To a solution of 5-nitro-1*H*-benzimidazole-2-thiol (5.12 mmol) in DMSO (15 ml) was added potassium carbonate (5.2 mmol). After 15 min. at 298 K, methyl iodide (7.68 mmol) was added drop wise. Upon disappearance of the starting material, as indicated by TLC, the resulting mixture was evaporated. The crude material was dissolved with EtOAc (60 ml), washed with water and brine, dried over MgSO_4_ and the solvent was evaporated *in vacuo*. The resulting residue was purified by column chromatography (EtOAc/hexane 3/7). The title compound was recrystallized from acetone at room temperature giving colourless crystals (M.pt: 475 K, yield: 47%).

### 6. Refinement

H atoms were located in a difference map and treated as riding with C–H = 0.96 Å and C–H = 0.93 Å for methyl- and aromatic-H, respectively. All hydrogen with *U*_iso_(H) = 1.5 *U*_eq_ for methyl-H and *U*_iso_(H) = 1.2 *U*_eq_ for aromatic-H.

### Crystal data

**Table:**

C_9_H_9_N_3_O_2_S	*F*(000) = 464
*M**_r_* = 223.25	*D*_x_ = 1.497 Mg m^−^^3^
Monoclinic, *P*2_1_/*c*	Mo *K*α radiation, λ = 0.71073 Å
Hall symbol: -P 2ybc	Cell parameters from 2772 reflections
*a* = 11.7213 (4) Å	θ = 2.5–29.6°
*b* = 11.8991 (4) Å	µ = 0.31 mm^−^^1^
*c* = 7.3025 (3) Å	*T* = 296 K
β = 103.523 (1)°	Block, colourless
*V* = 990.26 (6) Å^3^	0.42 × 0.31 × 0.26 mm
*Z* = 4	

### Data collection

**Table:**

Bruker X8 APEX diffractometer	2772 independent reflections
Radiation source: fine-focus sealed tube	2350 reflections with *I* > 2σ(*I*)
Graphite monochromator	*R*_int_ = 0.025
φ and ω scans	θ_max_ = 29.6°, θ_min_ = 2.5°
Absorption correction: multi-scan (*SADABS*; Bruker, 2009)	*h* = −16→16
*T*_min_ = 0.658, *T*_max_ = 0.746	*k* = −16→15
11751 measured reflections	*l* = −10→8

### Refinement

**Table:**

Refinement on *F*^2^	Primary atom site location: structure-invariant direct methods
Least-squares matrix: full	Secondary atom site location: difference Fourier map
*R*[*F*^2^ > 2σ(*F*^2^)] = 0.039	Hydrogen site location: inferred from neighbouring sites
*wR*(*F*^2^) = 0.116	H-atom parameters constrained
*S* = 1.07	*w* = 1/[σ^2^(*F*_o_^2^) + (0.0644*P*)^2^ + 0.2102*P*] where *P* = (*F*_o_^2^ + 2*F*_c_^2^)/3
2772 reflections	(Δ/σ)_max_ = 0.001
136 parameters	Δρ_max_ = 0.34 e Å^−^^3^
0 restraints	Δρ_min_ = −0.19 e Å^−^^3^

### Special details

**Table:**

Geometry. All e.s.d.'s (except the e.s.d. in the dihedral angle between two l.s. planes) are estimated using the full covariance matrix. The cell e.s.d.'s are taken into account individually in the estimation of e.s.d.'s in distances, angles and torsion angles; correlations between e.s.d.'s in cell parameters are only used when they are defined by crystal symmetry. An approximate (isotropic) treatment of cell e.s.d.'s is used for estimating e.s.d.'s involving l.s. planes.
Refinement. Refinement of *F*^2^ against all reflections. The weighted *R*-factor *wR* and goodness of fit *S* are based on *F*^2^, conventional *R*-factors *R* are based on *F*, with *F* set to zero for negative *F*^2^. The threshold expression of *F*^2^ > σ(*F*^2^) is used only for calculating *R*-factors(gt) *etc*. and is not relevant to the choice of reflections for refinement. *R*-factors based on *F*^2^ are statistically about twice as large as those based on *F*, and *R*- factors based on all data will be even larger.

### Fractional atomic coordinates and isotropic or equivalent isotropic displacement parameters (Å^2^)

**Table:**

	*x*	*y*	*z*	*U*_iso_*/*U*_eq_	
C1	0.37904 (11)	0.74865 (11)	0.42804 (17)	0.0319 (3)	
C2	0.21992 (11)	0.66649 (11)	0.29323 (17)	0.0315 (3)	
C3	0.13098 (12)	0.58951 (11)	0.2205 (2)	0.0385 (3)	
H3	0.1435	0.5126	0.2365	0.046*	
C4	0.02393 (12)	0.63119 (12)	0.1243 (2)	0.0388 (3)	
H4	−0.0373	0.5820	0.0752	0.047*	
C5	0.00707 (11)	0.74680 (12)	0.10023 (18)	0.0344 (3)	
C6	0.09247 (11)	0.82662 (11)	0.17081 (17)	0.0331 (3)	
H6	0.0793	0.9034	0.1538	0.040*	
C7	0.19868 (11)	0.78276 (10)	0.26849 (16)	0.0295 (3)	
C8	0.58418 (14)	0.64247 (14)	0.5740 (2)	0.0502 (4)	
H8A	0.6651	0.6472	0.6400	0.075*	
H8B	0.5788	0.6101	0.4518	0.075*	
H8C	0.5424	0.5961	0.6440	0.075*	
C9	0.32546 (14)	0.95396 (12)	0.3712 (3)	0.0487 (4)	
H9A	0.2561	0.9940	0.3088	0.073*	
H9B	0.3884	0.9716	0.3123	0.073*	
H9C	0.3470	0.9756	0.5013	0.073*	
N1	0.30262 (9)	0.83409 (9)	0.35759 (15)	0.0326 (2)	
N2	0.33342 (10)	0.64715 (10)	0.39349 (16)	0.0353 (2)	
N3	−0.10606 (11)	0.78650 (12)	−0.00931 (19)	0.0451 (3)	
O1	−0.17998 (12)	0.71648 (13)	−0.0807 (2)	0.0775 (5)	
O2	−0.12317 (11)	0.88741 (11)	−0.02760 (19)	0.0610 (3)	
S1	0.52130 (3)	0.78014 (3)	0.54830 (5)	0.04086 (13)	

### Atomic displacement parameters (Å^2^)

**Table:**

	*U*^11^	*U*^22^	*U*^33^	*U*^12^	*U*^13^	*U*^23^
C1	0.0322 (6)	0.0338 (6)	0.0290 (6)	−0.0034 (5)	0.0058 (4)	−0.0008 (5)
C2	0.0328 (6)	0.0299 (6)	0.0312 (6)	−0.0036 (4)	0.0066 (4)	−0.0010 (4)
C3	0.0407 (7)	0.0282 (6)	0.0445 (7)	−0.0073 (5)	0.0062 (5)	−0.0029 (5)
C4	0.0351 (6)	0.0374 (7)	0.0426 (7)	−0.0098 (5)	0.0062 (5)	−0.0089 (6)
C5	0.0290 (6)	0.0422 (7)	0.0312 (6)	−0.0004 (5)	0.0053 (5)	−0.0059 (5)
C6	0.0342 (6)	0.0306 (6)	0.0340 (6)	0.0001 (5)	0.0068 (5)	−0.0027 (5)
C7	0.0311 (6)	0.0291 (6)	0.0281 (5)	−0.0047 (4)	0.0067 (4)	−0.0022 (4)
C8	0.0382 (7)	0.0525 (9)	0.0537 (9)	0.0044 (6)	−0.0016 (6)	−0.0025 (7)
C9	0.0460 (8)	0.0291 (7)	0.0657 (10)	−0.0091 (6)	0.0024 (7)	−0.0022 (6)
N1	0.0318 (5)	0.0282 (5)	0.0356 (5)	−0.0054 (4)	0.0032 (4)	−0.0020 (4)
N2	0.0338 (5)	0.0316 (5)	0.0379 (5)	−0.0027 (4)	0.0033 (4)	0.0007 (4)
N3	0.0337 (6)	0.0560 (8)	0.0427 (6)	0.0032 (5)	0.0032 (5)	−0.0095 (5)
O1	0.0439 (7)	0.0760 (10)	0.0950 (11)	−0.0049 (6)	−0.0193 (7)	−0.0228 (8)
O2	0.0467 (6)	0.0567 (8)	0.0720 (8)	0.0149 (5)	−0.0012 (6)	−0.0006 (6)
S1	0.0342 (2)	0.0423 (2)	0.0412 (2)	−0.00665 (13)	−0.00108 (13)	−0.00503 (13)

### Geometric parameters (Å, º)

**Table:**

C1—N2	1.3210 (17)	C6—H6	0.9300
C1—N1	1.3730 (17)	C7—N1	1.3819 (15)
C1—S1	1.7342 (13)	C8—S1	1.7882 (17)
C2—N2	1.3796 (16)	C8—H8A	0.9600
C2—C3	1.3960 (17)	C8—H8B	0.9600
C2—C7	1.4098 (18)	C8—H8C	0.9600
C3—C4	1.379 (2)	C9—N1	1.4504 (17)
C3—H3	0.9300	C9—H9A	0.9600
C4—C5	1.395 (2)	C9—H9B	0.9600
C4—H4	0.9300	C9—H9C	0.9600
C5—C6	1.3888 (18)	N3—O2	1.2196 (18)
C5—N3	1.4578 (18)	N3—O1	1.2270 (18)
C6—C7	1.3841 (17)		
			
N2—C1—N1	113.95 (11)	S1—C8—H8A	109.5
N2—C1—S1	126.34 (11)	S1—C8—H8B	109.5
N1—C1—S1	119.71 (10)	H8A—C8—H8B	109.5
N2—C2—C3	129.35 (12)	S1—C8—H8C	109.5
N2—C2—C7	110.56 (11)	H8A—C8—H8C	109.5
C3—C2—C7	120.09 (12)	H8B—C8—H8C	109.5
C4—C3—C2	117.86 (12)	N1—C9—H9A	109.5
C4—C3—H3	121.1	N1—C9—H9B	109.5
C2—C3—H3	121.1	H9A—C9—H9B	109.5
C3—C4—C5	120.27 (12)	N1—C9—H9C	109.5
C3—C4—H4	119.9	H9A—C9—H9C	109.5
C5—C4—H4	119.9	H9B—C9—H9C	109.5
C6—C5—C4	123.99 (12)	C1—N1—C7	105.98 (10)
C6—C5—N3	117.79 (13)	C1—N1—C9	127.50 (11)
C4—C5—N3	118.20 (12)	C7—N1—C9	126.52 (12)
C7—C6—C5	114.64 (12)	C1—N2—C2	104.24 (11)
C7—C6—H6	122.7	O2—N3—O1	122.70 (14)
C5—C6—H6	122.7	O2—N3—C5	118.97 (13)
N1—C7—C6	131.58 (12)	O1—N3—C5	118.32 (14)
N1—C7—C2	105.28 (11)	C1—S1—C8	100.32 (7)
C6—C7—C2	123.13 (11)		

### Hydrogen-bond geometry (Å, º)

**Table:**

*D*—H···*A*	*D*—H	H···*A*	*D*···*A*	*D*—H···*A*
C8—H8*A*···O1^i^	0.96	2.53	3.393 (2)	150
C3—H3···O2^ii^	0.93	2.65	3.3038 (19)	128
C9—H9*A*···O2^iii^	0.96	2.67	3.563 (2)	155

Symmetry codes: (i) *x*+1, *y*, *z*+1; (ii) −*x*, *y*−1/2, −*z*+1/2; (iii) −*x*, −*y*+2, −*z*.
